# Headache as a predictor for dementia: The HUNT Study

**DOI:** 10.1186/s10194-015-0573-x

**Published:** 2015-10-15

**Authors:** Ane Karoline Stræte Røttereng, Ole Bosnes, Eystein Stordal, John-Anker Zwart, Mattias Linde, Lars Jacob Stovner, Knut Hagen

**Affiliations:** Department of Neuroscience, Norwegian University of Science and Technology, Trondheim, Norway; Department of Psychiatry, Namsos Hospital, Namsos, Norway; Norwegian Advisory Unit on Headache, St. Olavs University Hospital, 7006 Trondheim, Norway; Department of Neurology and FORMI, Oslo University Hospital, Oslo, Norway; Institute of Clinical Medicine, University of Oslo, Oslo, Norway

**Keywords:** Headache, Migraine, Dementia, Cognitive decline

## Abstract

**Background:**

The impact of headache on dementia is largely unknown. This study examined the association between headache and dementia using data from a large population-based study.

**Methods:**

This population-based study used data from the Nord-Trøndelag Health Surveys performed in 1995–1997 (HUNT2) and 2006–2008 (HUNT3). The reference group (controls) was participants aged ≥55 years who answered the headache questions in HUNT2 and later participated in HUNT3 (*n* = 15,601). The association with headache status in HUNT2 was investigated in sample of confirmed non-demented elderly evaluated with psychometric tests after HUNT3 (*n* = 96), and HUNT2 participants later diagnosed with dementia during 1997–2011 (*n* = 746). The association with headache was evaluated by logistical regression with adjustment for age, gender, level of education, comorbidity, smoking, and anxiety and depression.

**Results:**

Any headache was more likely to be reported in HUNT2 among those who later were included in the dementia registry (OR 1.24; 95 % CI 1.04–1.49) compared to the reference group, but less likely among the confirmed non-demented individuals (OR 0.62; 95 % CI 0.39–0.98). This relationship was even stronger for non-migrainous headache, whereas such association was not found for migraine.

**Conclusions:**

Compared to the reference group, individuals with dementia were more likely to report non-previous migrainous headache in HUNT2, whereas a sample of confirmed non-demented were less likely to report previous non-migrainous headache.

## Background

Headache is a common disorder affecting approximately 46 % of the adult population and is the third highest cause of disability worldwide [1, 2]. The prevalence of headache decreases distinctly after 50 years of age, but it is still a common complaint in the elderly population [3, 4]. Among middle-aged or elderly headache patients a large number of comorbid conditions have been identified, e.g., psychiatric disorders [5–8]. However, relatively few studies have evaluated the relationship between headache and cognitive status and dementia, and the majority of previous studies have focused on migraine [9–16]. Although a Swedish registry study reported an association between migraine and the occurrence of dementia with Lewy bodies [16], most other studies have not found any relationship between migraine and cognitive decline [9–15].

In a longitudinal Norwegian population-based study we have previously reported that headache at baseline was associated with slightly higher risk of dementia [17]. In that study, individuals not included in a dementia registry were classified as non-demented because they had managed to answer two questionnaires and a personal interview. No specific cognitive tests were performed. After that study was published, the dementia registry has been expanded with about 50 % more individuals, and in addition, we have new data including a group of definitely non-demented aged 55–89 years, verified by performance on tests of memory and intelligence.

The purpose of the present population-based study was to evaluate the association between primary headache and the development of dementia versus later belonging to a group of confirmed non-demented elderly, using a large group of health survey participants as reference group.

## Methods

### Study population

All inhabitants of Nord-Trøndelag county in Norway 20 years and older have been invited to three surveys, HUNT1 (1984–1986), HUNT2 (1995–1997), and HUNT3 (2006–2008) [18]. The overall participation rate has been 90 % for HUNT1, 70 % for HUNT2, and 54 % for HUNT3, respectively [18]. In all three surveys participants were asked to complete two extensive questionnaires (Q1 and Q2) including more than 200 health-related items, and they were also invited to a brief medical examination. Among respondents of Q1, approximately 80 % have also completed Q2 (i.e., 20 % Q2 dropouts).

The present study used data from HUNT2 and HUNT3.

**Fig. 1:**
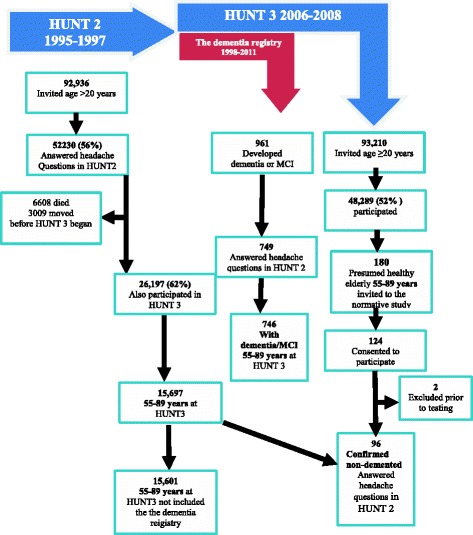
Flow diagram of the study population

Among 92,938 invited individuals in HUNT2, 64,787 (70 %) answered Q1, of whom 52,230 (56 %) answered the headache questions in Q2. Details of nonresponders have been described previously [3]. Individuals who responded to the headache questions were younger, were more likely to be women and had higher socioeconomic status than nonreponders [3]. Among responders, a total of 6608 had died and 3009 had moved from Nord-Trøndelag prior to HUNT3. Of 42,541 eligible, 26,197 (62 %) individuals also participated and completed all questionnaires in HUNT3. Among these, 15,697 were in the age interval 55–89 years at HUNT3 and therefore eligible for the present study (Fig. 1).

### The sample of confirmed non-demented

The sample was originally selected from the HUNT3 research Centre database for a separate study that originally had the aim to find a healthy Norwegian sample for testing translations of the Wechsler Memory Scale-III and the Wechsler Adult Intelligence Scale-III, to establish whether published American norms and principles for interpretation could be used in Norwegian clinical practice [19]. Based on information provided by the database, the HUNT research centre picked out 180 presumably healthy individuals, 30 in each age group (55–64, 65–69, 70–74, 75–79, 80–84 and 85–89 years old). The exclusion criteria were a) known disease in the central nervous system, b) known severe psychiatric illness c) known abuse of alcohol/other stimulants, d) severely impaired vision/hearing, e) impaired mobility to a degree that complicated execution of testing. Each invited individual was interviewed before testing, and asked questions about any mental or physical disease that might affect their cognition, or make it difficult to accomplish the testing. The sample therefore consists of presumably healthy individuals. Out of the 124 invited who consented to participate, a total of 122 persons (66 %) aged 57–89 years (M = 74.2; SD 8.8), with 7–21 years of education (M = 10.7; SD 3.2) were included. Of these, 52.5 % were women. In accordance with population data from Statistics Norway [20], the number of women was highest in the oldest age group, average education level lower in the oldest age group and lower for women than men in all age groups. Two individuals were excluded due to known degenerative brain organic illness revealed through questioning on health condition prior to the testing. Among the 122 evaluated with tests of memory and intelligence, 96 of confirmed non-demented had previously answered the headache questionnaire in HUNT2 (Fig. 1).

### Confirmed with dementia

This study includes data from the Dementia registry established through the Health and Memory Study (HMS), from which the dementia diagnoses were obtained. Data for the HMS were collected through two procedures. First, during the period 2008–2010, patient files from 1998 to 2010 in the two hospitals in Nord-Trøndelag county (Namsos Hospital and Levanger Hospital) were examined to identify those who had been registered with a diagnosis of dementia. Secondly, during 2010–2011, all inhabitants residing in nursing homes in Nord-Trøndelag were invited to participate in an extensive health examination focused on dementia diagnoses and related variables, using standardized interviews to assess cognitive decline and dementia. A total of 1332 dementia cases were identified: 104 from both hospital and nursing home files, 727 from only hospital files, and 501 from only nursing home files. The hospital cases were diagnosed by clinical experts in dementia, consisting of an internist (geriatrican) and two psychogeriatrists. The diagnoses were made according to national and international guidelines. More details of the distribution of dementia diagnoses identified in hospital are published elsewhere [17, 21]. The nursing home data were collected by nurses with adequate clinical experience [21]. In addition, two physicians with wide clinical and research experience independently diagnosed dementia subtypes using all available information. If the two physicians disagreed about diagnosis, a third expert was consulted to reach consensus [21]. The distribution of dementia diagnoses identified in nursing homes is published elsewhere [21].

The dementia registry was linked to data from the HUNT study using the Norwegian 11-digit personal identity number, which is unique to each resident in Norway. A total of 746 (56 %) had answered the headache questions in HUNT2 (Fig. 1), whereof 378 were identified from hospitals by a diagnostic hospital team [17], the remaining 368 by a nursing home team [21]. Because the diagnostic procedure and validity of diagnoses regarding dementia with Lewy body and frontotemporal dementia were somewhat different in the hospital and nursing home as described above, we have simplified the diagnostic categories of dementia into four different groups; Alzheimer’s disease (AD), vascular dementia (VaD), mixed dementia (AD + VaD), and lastly all other types of dementia (including dementia with Lewy body, frontotemporal dementia, and mild cognitive impairment).

### Headache diagnoses

Subjects who answered yes to the screening question “Have you suffered from headache during the last 12 months?” in Q2 were classified as having “any headache” and the remaining as headache free. Based on the subsequent 12 headache questions, headache sufferers were classified by applying modified migraine criteria of the International Classification of Headache Disorders, first edition (ICHD-I) [22]. Headache that did not fulfil the criteria for migraine was classified as non-migrainous headache. Migraine and non-migrainous headache were also categorized according to frequency (1–14 days or ≥ 15 days per month).

The validity of these questionnaire-based diagnoses has been reported previously [23]: for any headache, sensitivity was 85 % and specificity 83 % (kappa value 0.57); for migraine, sensitivity was 69 % and specificity 89 % (kappa value 0.59); and for non-migrainous headache, sensitivity was 61 %, specificity 81 % (kappa 0.43) [23]. In the validation study, 80 % of individuals with non-migrainous headache suffered from tension-type headache [23].

### Study design

In this population-based study the reference group were participants aged ≥55 years who answered the headache questions in HUNT2 and later participated in HUNT3. The reference group association was compared with HUNT2-participants who later were included in a group of confirmed non-demented elderly evaluated with psychometric tests after HUNT3, and with HUNT2 participants who later were diagnosed with dementia during 1997–2011.

### Potential confounders

In the HUNT2 data file the following seven variables were available: 1) Educational level (categorized according to duration: < 9 years, 10–12 years or > 13 years); 2) Anxiety and depression assessed by the Hospital Anxiety and Depression Scale (total HADS score); 3) Smoking (current, previous, or never); 4) Body mass index (BMI); 5) Systolic blood pressure (BP); 6) Physical activity categorized according to the response to two questions about duration and intensity of exercise per week (≥3 h’ hard physical activity, 1–2 h’ hard physical activity, ≥3 h’ light physical activity, 1–2 h’ light physical activity, or physical inactivity, 0 h); and 7) Presence of severe comorbidity based on the inclusion and exclusion criteria for individuals selected to the normative study of memory and intelligence 19] (severe visual and/or auditory problems, severe psychiatric problems, score of ≥ 1 on the CAGE questionnaire indicating possible alcohol overuse and/or self-reported stroke).

### Statistical analysis

We evaluated the association between headache in HUNT2 and later development of dementia or status of confirmed non-demented elderly by multivariate analyses using logistic regression with 95 % confidence intervals (CI). The analyses were initially adjusted for age and gender only. Subsequently, additional adjustments for other potential confounding factors were performed separately or together, but these factors were excluded from the final analyses if the OR changed less than 0.05. In the final multivariate analyses we adjusted for age (continuous variable), education (three categories), total Hospital and Anxiety and Depression (HADS) score (three categories), severe comorbid condition (two categories), and smoking (three categories). Other potential confounding factors evaluated, but not included in the final analyses were: BMI, smoking, Systolic BP and physical activity. Statistical analyses were performed with the Predictive Analytics Software (PASW) Statistics version 22 by SPSS Inc, an IBM Company (Chicago, IL, USA).

### Ethics

This study was approved by the Regional Committee for Ethics in Medical Research (REK Midt, St. Olavs Hospital, Trondheim). All HUNT2 and HUNT3 participants gave a written consent. A written consent was also given by those who were evaluated with psychometric tests after HUNT3, and individuals with dementia included from nursing home. Due to the retrospective design of dementia data collection from hospital, an informed consent for inclusion was not possible.

## Results

Characteristics of the study population are given in Table 1. Compared to the sample of confirmed non-demented, individuals who developed dementia were older, had higher systolic BP and total HADS score, had shorter education, and were more likely to be abstainers from alcohol (Table 1).

**Table 1:** Characteristics of the study population at baseline in HUNT2

	Confirmed non-demented	Dementia	HUNT3-participants aged 55–89 years
Participants	96	746	15,601
Women (%)	61.5	63.0	55.0
Mean age in years (SD)	61.8 (8.6)	71.3 (7.4)	56.3 (8.5)
Education >12 years (%) (missing = 106)	21.7	4.6	19.4
Current daily smoking (%) (missing = 11)	49.0	39.1	52.0
Mean body mass index (SD)	26.7 (3.2)	27.0 (4.2)	26.8 (3.8)
Mean systolic blood pressure (SD)	142.7 (17.7)	153.5 (23.0)	139.6 (19.9)
Mean total HADS^a^ score (SD)	6.6 (3.8)	8.5 (5.2)	7.9 (5.5)
Abstainers from alcohol (%) (missing = 14)	10.4	33.2	11.5
Severe comorbidity^b^ (%)	5.2	16.0	16.5
≥1 h hard physical activity (%) (missing = 214)	30.2	18.2	41.3

^a^HADS = Hospital Anxiety and Depression Scale

^b^Severe visual and/or auditive problems, severe psychiatric problems, score of ≥ 1 on the CAGE questionnaire indicating possible alcohol overuse, and/or self-reported stroke

From the dementia registry we identified 746 patients (470 women) who had answered the headache questions in HUNT2; 381 with AD, 121 with VaD, 69 with mixed dementia (VaD + AD), and 175 with other types of dementia.

In the final multivariate analyses, adjusted for gender, age, education, smoking, HADS score and severe comorbid conditions, any headache was more likely to be reported in HUNT2 among those who later were included in the dementia registry (OR 1.24; 95 % CI 1.04–1.49) compared to reference group, but less likely among the confirmed non-demented individuals (OR 0.62; 95 % CI 0.39–0.98) (Table 2). This relationship was even stronger for non-migrainous headache which was more likely to be reported in HUNT2 among those who later were included in the dementia registry (OR 1.49; 95 % CI 1.24–1.80) compared to the reference group, but less likely among the confirmed non-demented individuals (OR 0.50; 95 % CI 0.28–0.89) (Table 2). Such association was not present for migraine, in fact migraine was less likely among patients who developed dementia (OR 0.44; 95 % CI 0.22–0.77) compared to controls (Table 2). Concerning headache frequency, very few individuals with headache ≥ 15 days/month at baseline developed dementia (*n* = 21) or were included among the confirmed non-demented (*n* = 3). No clear relationship was found between headache ≥ 15 days/month and dementia or being confirmed non-demented (data not shown).

**Table 2:** Prevalence OR^a^ of any headache, migraine and non-migrainous headache related to status of confirmed non-demented or four different groups dementia

		Any headache		Migraine		Non-migrainous headache	
Total number	16,443	6673	OR (95 % CI)	2003	OR (95 % CI)	4670	OR (95 % CI)
Unselected HUNT-population	15,601	6409	1.0 (Ref.)	1970	1.0 (Ref.)	4439	1.0 (Ref.)
Confirmed non-demented	96	25	0.62 (0.39–0.98)	11	0.89 (0.46–1.72)	14	0.50 (0.28–0.89)
Dementia, all types	746	239	1.24 (1.04–1.49)	22	0.44 (0.28–0.70)	217	1.49 (1.24–1.80)
Alzheimer’s disease	381	112	1.07 (0.95–1.37)	11	0.40 (0.22–0–77)	101	1.29 (1.00–1.67)
Vascular dementia	121	46	1.56 (1.06–2.36)	7	0.97 (0.43–2.16)	39	1.74 (1.16–2.61)
Mixed dementia	69	25	1.70 (1.02–2.84)	3	0.79 (0.24–2.60)	22	1.99 (1.17–3.38)
Other types of dementia	175	56	1.24 (0.89–1.74)	1	0.09 (0.01–0.62)	55	1.60 (1.14–2.25)

^a^Adjusted for age, gender, level of education, severe comorbidity, smoking and total HADS score

Regarding diagnoses of dementia, any headache and non-migrainous headache were most clearly associated with the development of vascular dementia and mixed dementia (Table 2).

## Discussion

Headache, in particular non-migrainous headache, was more likely to be reported at baseline in HUNT2 among those who later developed dementia, when compared to the reference group. Conversely, headache and non-migrainous headache were less likely to be reported in HUNT2 among those who later were included in the sample of confirmed non-demented.

In our previous paper based on 378 cases with dementia, headache at baseline was associated with higher risk of dementia (HR 1.3, 95 % 1.1–1.7) when considering all individuals not included in a dementia registry as non-demented [17]. The present study included a larger number of dementia patients, and in addition a new subgroup of dementia-free documented by tests of memory and intelligence. The strongest association was found between non-migrainous headache and dementia (OR 1.5, 95 % 1.2–1.8). The opposite relationship was found between non-migrainous headache and the dementia-free group, whereas no clear relationship was found between migraine and dementia.

### Comparison with other studies

Few other registry studies have evaluated the association between headache and the development of dementia. An association between migraine and the occurrence of dementia with Lewy bodies was found in a study linking the Swedish dementia registry and the patient registry [16]. However, although the results in previous studies on migraine and cognitive decline have been somewhat mixed, results from longitudinal studies consistently have found that individuals with migraine are not at increased risk of cognitive decline [9–15]. However, to the best of our knowledge, no previous registry studies from other countries have evaluated the relationship between non-migrainous headache and dementia.

### Strengths and limitations of the study

The major strengths of this study are the population-based design and the use of validated headache diagnoses with good sensitivity and high specificity for both migraine and non-migrainous headache [23]. The diagnoses of dementia were verified in all patients by a team of clinical experts in dementia. However, because the diagnostic procedure differed in cases identified by the hospital and nursing home, a limited number of specific dementia diagnoses were used in the present study. The group of confirmed non-demented underwent a medical examination and were interviewed about their health prior to the cognitive testing. In the multivariate analyses, we were able to adjust for a large number of potential confounding factors. Nevertheless, the possibility of residual confounding by unrecognized factors cannot be wholly excluded.

There are also several study limitations that should be considered. First, despite the large sample size, the group of definite non-demented was relatively small. It should be emphasized that a wide 95 % confidence intervals was found for several analyses, and the results for these analyses should be interpreted with caution, in particular regarding migraine. Second, although 62 % of eligible subjects participated in HUNT3, a selection bias cannot be ruled out. Since the response rate of the headache questions in HUNT2 was 56 % of the total adult population in Nord-Trøndelag, one may question to what degree the results can be generalized. Those who responded to the headache questions were younger, were more likely to be women and had higher socioeconomic status than nonresponders On the other hand, the wide scope of the HUNT studies makes participation bias with specific relevance to headache less likely.

### Interpretation

As stated in our previous paper [17], there are several ways to interpret that any headache, and in particular non-migrainous headache, were more likely to be reported at baseline among those who developed dementia.

Firstly, we cannot rule out that for some individuals early or presymptomatic condition of dementia was causing headache, and not headache that later caused dementia. Since all had managed to answer two questionnaires in HUNT2, it is not likely that any of them had developed a dementia at this time. However, some degree of mild cognitive impairment in some participants cannot be ruled out. For a total of 38 patients the interval between reporting headache and the diagnosis of dementia was less than five years. However, the mean interval until diagnosis of dementia was nine years.

Secondly, headache and dementia may share an underlying causal factor, e.g., cardiovascular risk factors or alcohol overuse [24–28]. Even though non-migrainous headache stood out as an independent associated factor for dementia when adjusted for available environmental factors, one cannot exclude the possibility for other shared environmental factors or a shared underlying genetic susceptibility [17].

Thirdly, it may be that headache is a true risk factor for vascular dementia. It is of relevance that headache status and the outcome “dementia” came from separate registries, and knowledge of a relationship between dementia and headache was missing when the dementia registry was established. If headache is indeed a true risk factor for dementia, the underlying mechanisms must be elucidated. Furthermore, adding headache questions in the clinical evaluation of cognitive function should also be considered.

In the present study we found that headache and non-migrainous headache were less likely to be reported among those who later were included among confirmed non-demented. We cannot rule out that this relationship may be artificial. Although pain or headache in particular were not among exclusion criteria for selection of this group, it may be that at least some individuals did not want to participate in the memory and intelligence test due to severe headache complaints that could cause reduced test results [29]. In addition, it should be emphasized that only presumed healthy individuals without severe comorbidity were invited to participate in the memory and intelligence test. Although we adjusted for presence of severe comorbidity in the multivariate analyses, the lower occurrence of non-migrainous headache in HUNT2 among those who later were confirmed to be non-demented could possibly be a consequence of lower prevalence of other health problems.

No statistical significant association was found between migraine and dementia in the present study. In accordance, most longitudinal studies consistently have found that individuals with migraine are not at increased risk of cognitive decline [9–15]. This knowledge should give reassurance to migraine sufferers who fear that their attacks could represent a risk factor of dementia in the long run.

## Conclusions

Any headache and non-migrainous headache were more likely to be reported at baseline among those who later were included in a dementia registry. There was no clear association between migraine and dementia. The sample of confirmed non-demented were less likely to report non-migrainous headache beforehand.

## Abbreviations

HUNT: The Nord-Trøndelag Health Study
AD: Alzheimer’s disease
VaD: Vascular dementia
ICHD: International Classification of Headache Disorders
BMI: Body mass index
HADS: Hospital Anxiety and Depression Scale
BP: Blood pressure
OR: Odds ratio
HR: Hazard Ratio
